# Molar concentration from sequential 2-D water-window X-ray ptychography and X-ray fluorescence in hydrated cells

**DOI:** 10.1038/srep24280

**Published:** 2016-04-12

**Authors:** M. W. M. Jones, K. D. Elgass, M. D. Junker, M. D. de Jonge, G. A. van Riessen

**Affiliations:** 1 Australian Synchrotron, 800 Blackburn Rd, Clayton, 3168, Australia; 2 ARC Centre of Excellence for Advanced Molecular Imaging, La Trobe Institute for Molecular Sciences, La Trobe University, Bundoora, 3086, Australia; 3 Monash Micro Imaging, Hudson Institute of Medical Research, 27-31 Wright Street, Clayton, 3168, Australia; 4 Department of Chemistry and Physics, La Trobe Institute for Molecular Science, La Trobe University, Victoria 3086, Australia

## Abstract

Recent developments in biological X-ray microscopy have allowed structural information and elemental distribution to be simultaneously obtained by combining X-ray ptychography and X-ray fluorescence microscopy. Experimentally, these methods can be performed simultaneously; however, the optimal conditions for each measurement may not be compatible. Here, we combine two distinct measurements of ultrastructure and elemental distribution, with each measurement performed under optimised conditions. By combining optimised ptychography and fluorescence information we are able to determine molar concentrations from two-dimensional images, allowing an investigation into the interactions between the environment sensing filopodia in fibroblasts and extracellular calcium. Furthermore, the biological ptychography results we present illustrate a point of maturity where the technique can be applied to solve significant problems in structural biology.

Biological X-ray Coherent Diffractive Imaging (CDI)^1^,^2^,^3^,^4^, in a multitude of guises, including Fresnel CDI^5^,^6^, phase-diverse CDI^7^,^8^, and ptychography^9^, has recently been the focus of significant research interest, mostly due to the ability to generate quantitative high-resolution, artefact free images of weakly scattering specimens with high natural contrast^4^,^9^. Scanning CDI methods, herein referred to generally as ptychography, allow specimens larger than the extent of a single X-ray probe to be imaged with fewer artefacts^10^,^11^,^12^, while providing directly quantitative information about the specimen^1^,^8^,^13^,^14^. Current ptychography techniques have demonstrated that artefacts can be suppressed virtually entirely^15^,^16^,^17^,^18^, with any artefacts remaining in cellular ptychography due to the preparation of the specimen rather than inherent to the technique^19^. To realise the potential of X-ray ptychography for biological applications, it is therefore necessary to avoid the structural and imaging artefacts caused by drying and or freezing^20^,^21^. The ubiquitous presence of water in biological systems suggests that scattering and absorption X-ray imaging should be applied using X-rays with energy in the range bound by the C and O K absorption edges that is known as the water window and where high natural phase^22^ and absorption^23^ contrast between protein and water occurs. However, even in the water window, significant absorption occurs limiting the specimen thickness, favouring the use of phase contrast at higher energies with thick specimens. Previously, ptychography has been demonstrated in the X-ray water window^24^,^25^ and for frozen specimens^21^,^26^, while X-ray CDI has been demonstrated for room-temperature hydrated specimens at hard X-rays^27^, however, hydrated room-temperature biological ptychography in the X-ray water-window has yet to be demonstrated.

It was recently shown that simultaneous X-ray ptychography/X-ray fluorescence microscopy (XFM), enables determination of structural information that is not accessible from XFM alone^21^. The resolution of X-ray fluorescence measurement can be improved beyond the probe size through deconvolution of the elemental maps by the probe function that was retrieved from the ptychography data^28^. These possibilities have led some to thinking that the simultaneous measurement is necessarily the optimum approach for biometals investigations.

The simultaneous measurement has further advantages: only one access to the beamline is required; there is no requirement to align two independent images; the sample cannot change between measurements. However, there is one significant drawback of the simultaneous measurement: the photon energy cannot be optimised for both techniques simultaneously, leading to a 100-fold decrease in sensitivity for the ptychographic data.

Soft X-ray fluorescence microscopy is limited by low fluorescence yield and high levels of self-absorption of low-*Z* elements, and so XFM is typically conducted at an incident energy greater than 10 keV, exciting the k-shell electrons of many biologically relevant elements from P (Z = 15) to Zn (Z = 30)^29^. The position of the k_α_ lines for a selection of these elements is shown in Fig. 1. However, for incident photon energy over 10 keV, weak absorption and phase contrast between biological material and water is unfavourable for ptychography. Figure 1 indicates that a 100-fold increase in phase sensitivity is achieved for hydrated biological specimens in the X-ray water window compared to 10 keV, essential for imaging fine biological features in hydrated environments. It is therefore clear that for hydrated biological specimens where fine features need to be resolved that sequential measurements optimised at two distinct incident energies is highly beneficial compared to a simultaneous measurement. As the X-ray energy decreases, the value of the complex scattering factor increases. While this allows for thin specimens to be imaged with high sensitivity, it also contributes to an increase in radiation dose for a given imaging time. However, Howells *et al.* (2009) have shown that visualising protein through X-ray diffraction microscopy at fixed resolution within a hydrated environment in the water window (284 eV–540 eV) delivers an order of magnitude less radiation dose than at X-ray energies above 2.5 keV^22^.

Hydrated-specimen water-window X-ray ptychography has the potential to probe the link between nanoscale cellular structure and the presence and concentration of specific elements. This is fundamental in understanding the cellular migration process^32^,^33^. Cells migrate through several actin rich structures, which at the cells leading edge of fibroblasts comprise of lamellipodia and filopodia. The lamellopodium is a flat protrusion around 100 to 200 nm thick containing a network of filament (F)-actin^32^. Filopodia extends from the lamellopodium in tight bunches of F-actin with a diameter of between 100 and 300 nm^32^. One of the roles of the filopodia is sensing and reacting to the environment to steer the migrating cell^33^,^34^. Intra- and extra-cellular Ca is known to play a role in the movement of fibroblasts by altering the direction of the filopodia^33^,^34^,^35^,^36^,^37^. Using a variety of methods, it has been shown that the angle the filopodia bend at in response to Ca is directly linked to the concentration of Ca^33^,^35^.

Here we present the first X-ray ptychography images of a hydrated unfrozen cellular specimen in the water-window. We combine the information from sequential X-ray ptychography and XFM measurements to investigate cellular migration. We use the high-resolution and high-sensitivity quantitative information about the hydrated specimen only obtainable through water-window ptychography to calculate the scattering quotient of areas of the specimen. This calculation allows us to determine the specimen thickness, and therefore to convert the X-ray fluorescence areal density to biologically relevant molar concentration. We use this technique to directly determine the influence of local extracellular Ca concentration, [Ca], on the bend angle of filopodia, which is problematic in a simultaneous measurement due to reduced sensitivity.

## Results and Discussion

### Soft X-ray Ptychography

Mouse Embryonic Fibroblasts (MEFs) were grown on silicon nitride membranes for 4 hours before being fixed in 2.5% glutaraldehyde and sealed in a fully hydrated environment. The specimens were then and imaged at room temperature using the Soft X-ray Imaging (SXRI) beamline at the Australian Synchrotron with an incident photon energy of 520 eV. MEFs are migrating cells, and as such have very fine features such as filopodia which they use to sense the surrounding environment and guide their movement. Filopodia are between 100 and 300 nm thick^32^, and have not previously been seen in X-ray diffraction measurements, most likely due to harsh preparation methods^19^. However, by sealing the cells in a fully hydrated environment, these fine features can be easily seen, as shown in Fig. 2. Also visible is the thin lamellopodium (L) and actin bundles (A). With the high level of image contrast and resolution, together with the absence of visible artefacts of any type we can readily see the filopodia as they bent and moved on the substrate. We note that although we are not able to visualise artefacts, the combination of chemical fixation and radiation dose would have resulted in some specimen damage, however, radiation-induced damage is not visible at this imaging resolution. Quantitative analysis of the phase and magnitude of the complex transmission function reveal that the MEF is immersed in approximately 2 μm of water, while both the filopodia and the lamellopodium are in the order of 100–300 nm thick, assuming their composition is close to model protein.

On close inspection, it can be seen that the bends in the filopodia are often adjacent to small X-ray dense areas on the substrate. The scattering quotient, *f*_*q*_, which is characteristic of composition is expressed as^19^


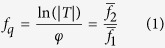


where T and φ are the transmission and phase retardation of the complex transmission function, and *f*_1_ and *f*_2_ are the real and imaginary components of the complex scattering factor. In the present case, maps of the scattering quotient of the aggregates can be obtained from the complex transmission function after subtracting the contribution from a thickness of water which is assumed to be approximately uniform over the area imaged. The scattering quotient map from aggregate (b) in Fig. 2 and are presented in Fig. 3. In this case, we obtain values of the scattering quotient for the filopodia and the aggregate of approximately 0.20. From this, we can deduce that both the filopodia and aggregates are a similar material that is a mix of proteins and lipids which have scattering quotients of between 0.16 and 0.26^18^,^19^. To simplify the analysis of the data, we have used a model protein with chemical formula equal to C_30_H_50_N_9_O_10_S and density of 1.35 gcm^2^ 22 as the composition.

### X-ray Fluorescence

The region of interest was then investigated at the nanoprobe of the X-ray Fluorescence Microscopy (XFM) beamline at the Australian Synchrotron^38^ using an incident energy of 10.1 keV without disturbing the hydrated cellular environment. In migrating cells, a global Ca gradient from the trailing to leading edge of the cell^33^,^39^ and an increased P concentration in the leading edge^39^ indicate the direction of motion. Figure 3A,B show the Ca and P fluorescence map of the entire MEF. The direction of migration, as indicated with an arrow, is evident from the Ca gradient, while the P distribution identifies the leading edge. It is known that chemical fixation methods can result in elemental redistribution, however in this case we see a clear correlation between the extracellular Ca distribution and the filopodia bend angle, a correlation that has no other credible cause. We therefore surmise that the extracellular Ca was not redistributed in the fixation process.

A second XFM image was taken in the area of interest at higher resolution and sensitivity to more thoroughly investigate the interaction between the extracellular Ca and the filopodia. The Ca distribution from this image is shown in Fig. 4C, overlaid with the phase of the complex transmission function initially presented in Fig. 2. In this image we can clearly see that many of the biological aggregates on the substrate contain significant amounts of Ca. There is also a region of high level Ca in an area within the lamellopodium which, combined with the structural information presented in Fig. 2, appears to be a focal adhesion site, indicated in Fig. 4C with an arrow. These regions are flat elongated structures that form the cell-substrate adhesions, and contain a large number of adhesion and signalling molecules including Ca^32^.

### Molar Concentration from 2D X-ray Fluorescence Mapping

Areas in Fig. 4C where Ca is co-localised with biological aggregates on the substrate allow the information from both ptychography and XFM images to be combined. As no other material is observed in the immediate vicinity in the ptychography image, the Ca in these areas can be assumed to have originated wholly from the aggregates. Figure 5A,B,D,E show the phase and Ca areal density of two aggregates which contain Ca and are associated with filopodia bends (i and ii in Fig. 4C), confirming the co-localisation of these two features.

Knowing the average composition of the Ca-rich aggregates, we can represent the phase information as thickness, and is shown as a surface plot in Fig. 5C,F with the areal density of Ca displayed as the colour information. Using this representation, we can determine the volume of material (*V*) that gives rise to the total amount of measured Ca in moles (*n*), and combine the information of the two imaging modalities to arrive at a molar concentration (*c*) of Ca in each aggregate; 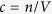. In the case of this measurement, the resolution of the two techniques is different, and the Ca map has been resampled to the resolution of the phase image.

Some of these aggregates, such as those presented in Fig. 5, are adjacent to distinct bends in filopodia, allowing the angle of the bend to be associated with a [Ca]. The results of this analysis are presented in Fig. 6, and show a strong correlation between the filopodia bend angle and the extracellular [Ca], supporting previous studies that showed the same relationship as a function of total accumulated Ca^35^,^36^. A single outlier is presented in Fig. 6 (^*^): In this case, the filopodia – identified in the inset of Fig. 2 as example (iii) – appears to have turned very sharply and then stopped growing – a fundamentally different response to the rest of the specimen. We note that the fluorescence data did not show anything different about this region, and can only hypothesize that it must be chemically rather than elementally different from the other regions.

## Conclusion

**Figure 1:**
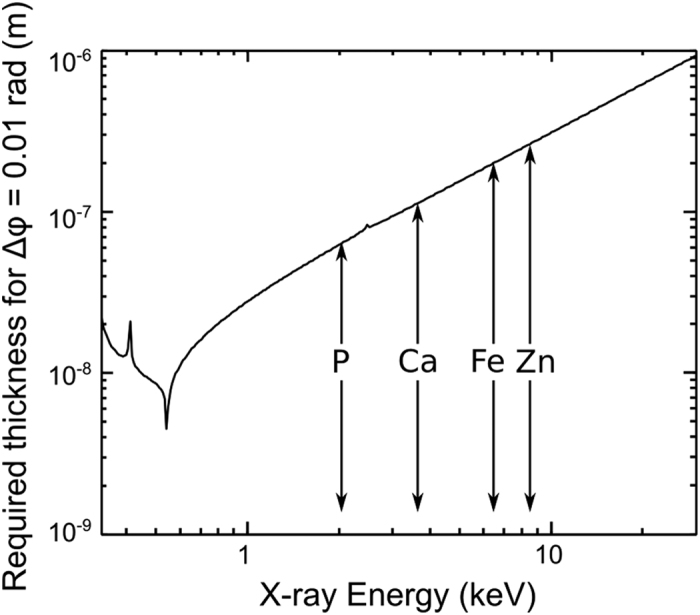
Required thickness of protein (C_30_H_50_N_9_O_10_ S and density of 1.35 gcm^2^)^22^ in water to induce a phase change of more than 0.01 radians greater than the surrounding water. For photon energy of 500 eV, the required thickness is 100 times less than for 10 keV, allowing 100 times greater sensitivity to fine features. The k_α_ line position for a selection of biologically relevant elements is also included (arrows). Figure prepared using data from^22^,^31^.

**Figure 2:**
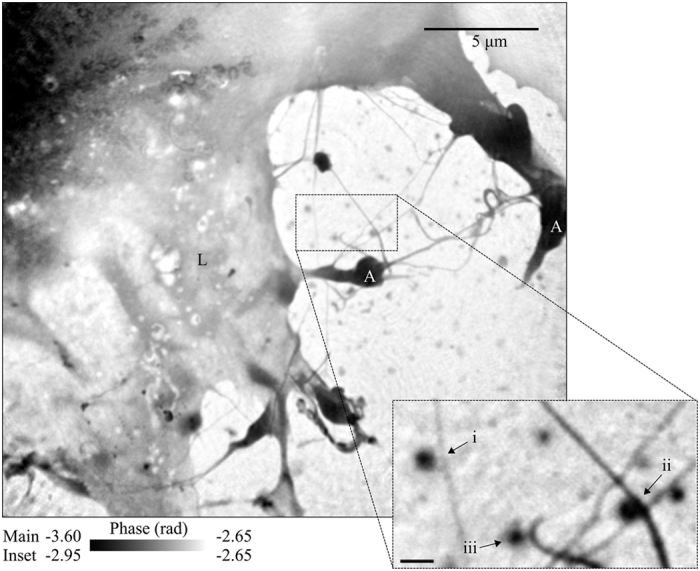
Phase of the complex transmission function of one corner of an MEF sealed in a fully hydrated environment. Here we see an area of the lamellopodium (L) with both actin bundles (A) and fine filopodia extending from it. Small aggregates of material can be seen in the extracellular regions. Inset shows bent filopodia co-located with small aggregates, labelled i to iii. The scale bar on the inset is equal to 500 nm.

**Figure 3:**
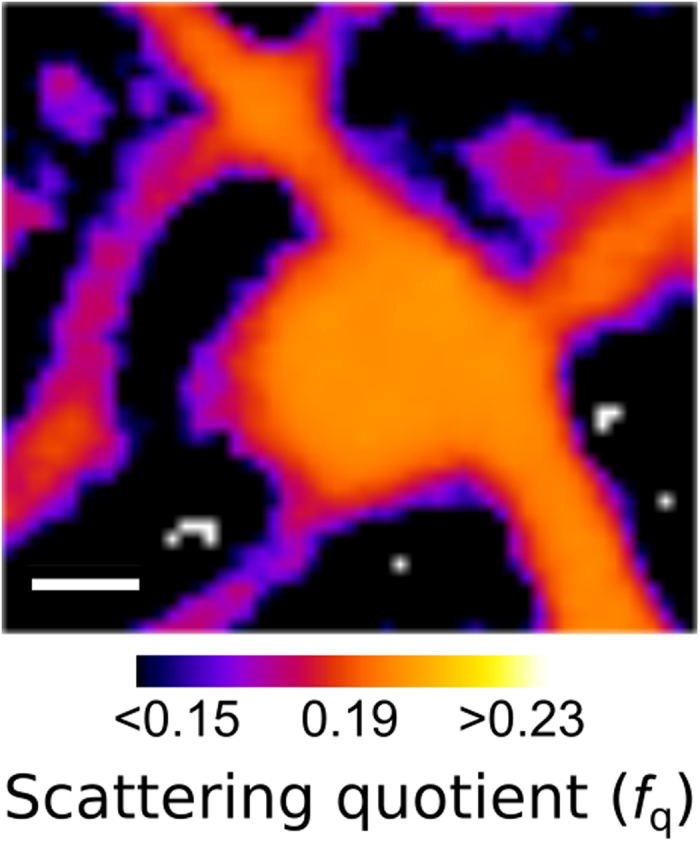
The scattering quotient map of aggregate (b) and filopodia identified in Fig. 2 (ii - inset). Here we can see that the scattering quotient of the aggregate and filopodia is approximately equal to 0.20, corresponding to a mix a protein and lipids. Scale bar equals 200 nm.

**Figure 4:**
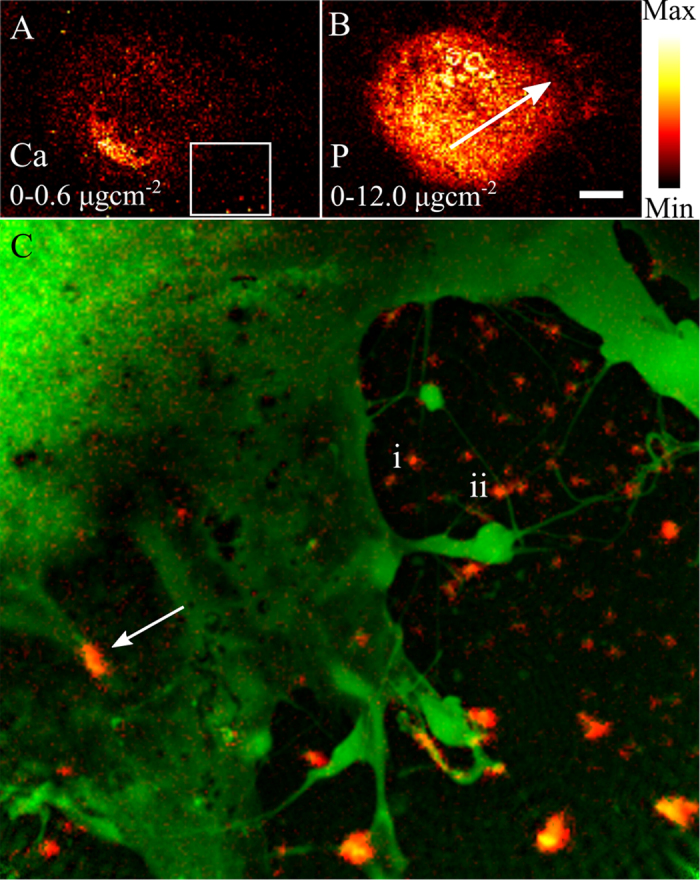
(**A**) Calcium and (**B**) Phosphorus X-ray fluorescence distributions of the entire MEF. As discussed in the text, these elemental distributions allow the direction of motion to be deduced, and is indicated in (**B**) with an arrow. The box in (**A**) indicated the region of interest, presented in (**C**), which shows the Ca distribution (red) overlaid with the phase of the complex transmission function of the same area initially presented in Fig. 2. The co-localisation of some features such as focal adhesion sites (arrow) and in extracellular features (lines) in both images is evident. In some case, the co-localisation of Ca and extracellular aggregates occurs at a bend in the filopodia (examples labelled i and ii correspond to those identified in Fig. 2). The scale bar in (**B)** is equal to 15 μm, while the areal density in μgcm^−2^ is given in each panel.

**Figure 5:**
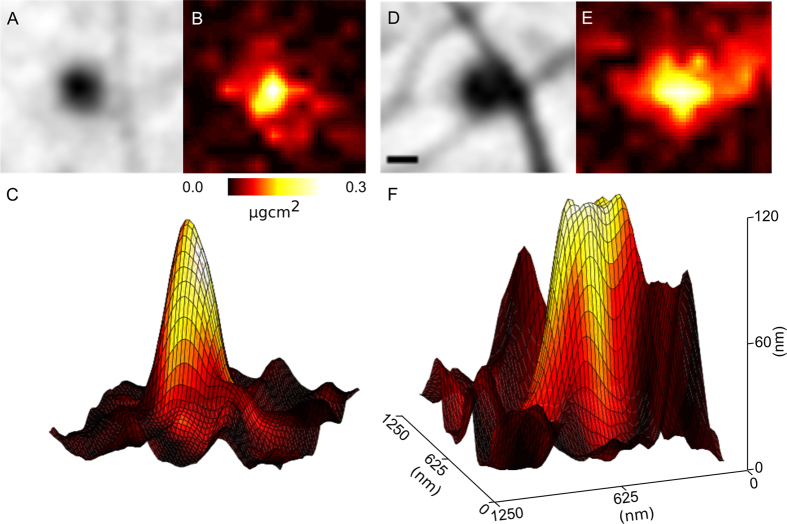
(**A**,**B**,**D**,**E**), phase and areal density of Ca of aggregates of biological material at bent filopodia identified in Figs 2 and 3 as i and ii. (**C**,**F**) Represent the calculated thickness of the biological aggregate with the colour of the surface mapped to the areal density of Ca (**B**,**E**). From these images, we can see the volume of biological material that gives rise to the Ca signal, and use this information to determine the molar concentration of Ca in each aggregate. The scale bar in A is equal to 200 nm, while the vertical axis in (**C**) refers to the thickness of protein in (**C**,**F**).

**Figure 6:**
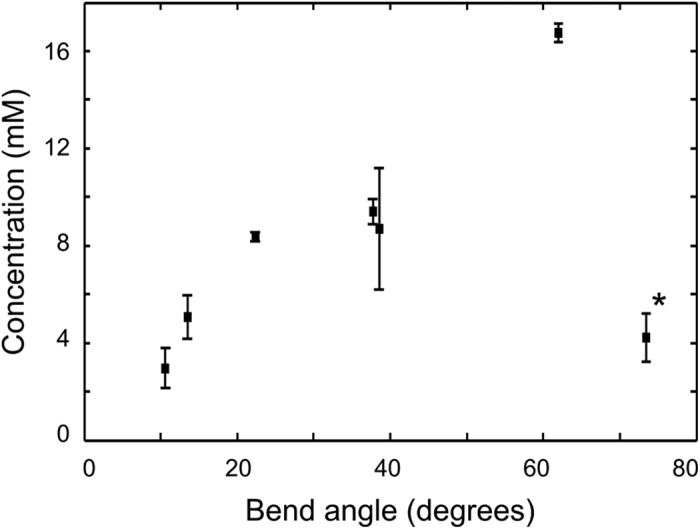
Relationship between filopodia bend angle and Ca concentration showing an increase in the bend angle as a function of [Ca]. This result is in line with previous studies^35^,^36^, demonstrating the effectiveness of the presented method.

We have imaged a MEF in a fully hydrated state using X-ray ptychography in the water-window. These first hydrated biological ptychography images in the water-window show outstanding quality, free from preparation and imaging artefacts, with high contrast between biological material and the surrounding water. We used the quantitative phase and magnitude values obtained from the complex transmission function though X-ray ptychography to extend complimentary X-ray fluorescence imaging. In this case, we use this information to determine the volume of material that a fluorescent signal originates from, and convert a projected two-dimensional areal elemental density into molar concentration. This added information allows biological questions to be addressed in ways that would not otherwise be possible. The method we describe can be used in either as two individual measurements as describe here, or in a simultaneous X-ray fluorescence and ptychography arrangement. Furthermore, we highlight that while simultaneous X-ray fluorescence and ptychography can offer significant advantages in some cases, in other cases, sequential mapping is the only way to achieve the desired result. For example, as we outline in Fig. 1, accessing fine hydrated biological features (E < 1 keV) and k-shell Ca fluorescence (E = 4038 eV) requires two mutually exclusive incident X-ray energies.

## Methods

### Cell Culture and Microscopy

Silicon nitride windows were coated with Poly-L-lysine (Sigma) for 2 h at 80 degrees, then dried overnight. MEF cells were cultured in complete DMEM with 10% FCS at 37 degrees and 5% CO_2_. To transfer cells onto silicon nitride windows, cells were trypsinized and then resuspended. 20 μl of the cell suspension was added on top of the Poly-L-lysine coated silicon nitride window and put back in the incubator for 4 h to let the cells attach to the window. Every 30 min the liquid was topped up with 10 μl fresh DMEM to prevent cells from drying out. Afterwards cells were fixed with 2.5% Glutaraldehyde and three times washed with distilled water. Chemical fixation in this way provides significant protection from X-ray radiation damage on the length scales we address^1^, while having only a minor effect on the X-ray fluorescence results^40^. Excess water was removed and a second silicon nitride window was placed upside down onto the window with cells, then the two windows were sealed with polyester resin and left for 24 hours for the resin to harden thoroughly. Sealed samples were stored at room temperature until imaging.

### X-ray Diffraction measurements

Ptychography data was collected at the Soft X-ray Imaging beamline (SXRI) at the Australian Synchrotron^41^. 520 eV X-ray photons were focused using a Fresnel zone plate (FZP) with a diameter of 300 um and an outer zone of 38 nm with a focal length of 4.8 mm. A combination of a 10 μm order sorting aperture (OSA) and a 30 μm beamstop eliminated all but the first order focus. A cooled charge-coupled device (CCD) detector (Princeton MT-MTE) with 2048 × 2048 square pixels each with 13.5 μm side length was placed 32 cm downstream of the FZP. Under this arrangement, the theoretical resolution limit due to the numerical aperture (NA) of the detector is equal to 28 nm.

Diffraction data frames was collected as individual 0.25 sec frames in a 4 × 4 raster scan with 1.5 μm grid spacing at two defocussed distances, defined as the distance between the focus of the FZP and the sample, of 270 and 375 μm. Six frames were discarded, and the resulting 26 frames had a minimum lateral overlap fraction between adjacent frames equal to 86%. The total imaging time was equal to 6.5 seconds, with an approximate imaging dose of 3.5 × 10^8^ Gy delivered to the sample, assuming the average composition of the sample is close to model protein, similar to the dose delivered in hydrated specimens without chemical fixation^27^ and freeze dried specimens^2^,^4^. The quantitative complex transmission function of the sample was obtained using 500 iterations of phase-diverse CDI reconstruction algorithms^12^ using a complex constraint^18^ as implemented in the NADIA software library (http://cxscode.ph.unimelb.edu.au).

### X-ray fluorescence measurements

Fluorescence data was collected using a Vortex detector at the X-ray Fluorescence Microscopy (XFM) beamline at the Australian Synchrotron^38^. 10.1 keV X-ray photons were focused using a FZP with a diameter of 160 μm to a focal spot approximately 250 nm. An overview scan was completed with a scan area of 100 × 85 μm with a step size of 0.86 μm (0.75 in *x* to account for the angle) and a dwell of 1 second. A second scan of the area of interest was completed with a 32 × 32 μm scan area with 130 nm (111 nm in *x* to account for the angle) step size and 1.5 second dwell. The total radiation dose delivered through XFM measurements was approximately 4.5 × 10^8^ Gy, yielding a total accumulated dose of approximately 8.0 × 10^8^ Gy. This total accumulated dose is comparable to previous studies with similar preparations where features were observed at 50–60 nm^1^. Data was analysed using MAPS^42^, and exported as 32-bit images for analysis.

### Molar concentration determination

In this proof of principle experiment, the fluorescence and ptychography images were manually registered after the fluorescence image was resampled to the same pixel pitch as the ptychography data. The molar concentration of Ca was then calculated from the volume of the biological aggregate determined through the ptychography image, and the total amount of Ca in each aggregate determined from the fluorescent image. By determining the average composition of the material through the calculation of the scattering quotient, and combining the information obtained by this measure with the quantitative phase measurement of the sample, the volume of each aggregate was calculated. The amount of Ca in each aggregate was calculated by using the quantitative information obtained through the MAPS analysis combined with the area of each aggregate. The molar concentration was then calculated and plotted against the measured bend angle. Error bars contain errors propagated from MAPS analysis (<5%), phase images (0.01 rad), and registration and measurement of the aggregate areas and thickness which contribute to errors in both the aggregate area and position, and therefore in the reported Ca concentration.

## Additional Information

**How to cite this article**: Jones, M. W. M. *et al.* Molar concentration from sequential 2-D water-window X-ray ptychography and X-ray fluorescence in hydrated cells. *Sci. Rep.* **6**, 24280; doi: 10.1038/srep24280 (2016).
